# Echocardiographic assessment of myocardial function and mechanics during veno-venous extracorporeal membrane oxygenation

**DOI:** 10.1530/ERP-18-0071

**Published:** 2019-04-03

**Authors:** David G Platts, Kenji Shiino, Jonathan Chan, Darryl J Burstow, Gregory M Scalia, John F Fraser

**Affiliations:** 1 Department of Echocardiography, The Prince Charles Hospital, Brisbane, Queensland, Australia; 2 Critical Care Research Group, The Prince Charles Hospital, Brisbane, Queensland, Australia; 3 The University of Queensland, Brisbane, Queensland, Australia; 4 School of Medicine, Menzies Health Institute Queensland, Griffith University, Gold Coast, Queensland, Australia; 5 Fujita Health University, Toyoake, Japan; 6 Adult Intensive Care Service, The Prince Charles Hospital, Brisbane, Queensland, Australia

**Keywords:** transthoracic echocardiography, contrast-enhanced echocardiography, speckle tracing echocardiography, extracorporeal membrane oxygenation

## Abstract

**Background:**

Transthoracic echocardiography (TTE) plays a fundamental role in the management of patients supported with extra-corporeal membrane oxygenation (ECMO). In light of fluctuating clinical states, serial monitoring of cardiac function is required. Formal quantification of ventricular parameters and myocardial mechanics offer benefit over qualitative assessment. The aim of this research was to compare unenhanced (UE) versus contrast-enhanced (CE) quantification of myocardial function and mechanics during ECMO in a validated ovine model.

**Methods:**

Twenty-four sheep were commenced on peripheral veno-venous ECMO. Acute smoke-induced lung injury was induced in 21 sheep (3 controls). CE-TTE with Definity using Cadence Pulse Sequencing was performed. Two readers performed image analysis with TomTec Arena. End diastolic area (EDA, cm^2^), end systolic area (ESA, cm^2^), fractional area change (FAC, %), endocardial global circumferential strain (EGCS, %), myocardial global circumferential strain (MGCS, %), endocardial rotation (ER, degrees) and global radial strain (GRD, %) were evaluated for UE-TTE and CE-TTE.

**Results:**

Full data sets are available in 22 sheep (92%). Mean CE EDA and ESA were significantly larger than in unenhanced images. Mean FAC was almost identical between the two techniques. There was no significant difference between UE and CE EGCS, MGCS and ER. There was significant difference in GRS between imaging techniques. Unenhanced inter-observer variability was from 0.48–0.70 but significantly improved to 0.71–0.89 for contrast imaging in all echocardiographic parameters.

**Conclusion:**

Semi-automated methods of myocardial function and mechanics using CE-TTE during ECMO was feasible and similar to UE-TTE for all parameters except ventricular areas and global radial strain. Addition of contrast significantly decreased inter-observer variability of all measurements.

## Introduction

Extra corporal membrane oxygenation (ECMO) is a specialised form of pulmonary or cardiopulmonary support in critically unwell patients (1, 2, 3). In light of the nature of these patients and their dynamic course, echocardiography plays an important role in monitoring ventricular function during ECMO (4, 5, 6, 7, 8). However, patients supported with ECMO are typically in the critical care complex and there are several adverse factors that can diminish the quality of transthoracic echocardiographic (TTE) images. It is well recognised that contrast-enhanced transthoracic echocardiographic imaging can improve technically difficult echocardiographic studies within the critical care complex (9, 10, 11, 12, 13, 14, 15, 16).

Assessment of ventricular function ranges from a qualitative visual evaluation to more formal quantification such as volumes, ejection fraction and tissue Doppler parameters (17, 18). More recently, speckle tracking echocardiography (STE) has become integrated into the clinical practice for the assessment of clinical and pre-clinical myocardial dysfunction. STE is a form of imaging that tracks the motion of pre-defined speckles within the myocardium throughout the cardiac cycle. As a result of this tracking, information is obtained about myocardial mechanics or deformation (19, 20, 21). However, the accuracy of STE when combined with contrast-enhanced TTE has not been clearly defined. Adequate image quality is typically required for STE to be feasible and accurate. Sub-optimal images, which can be rendered adequate with contrast, have traditionally not been evaluated using STE due to the perception of difficulty in tracking the speckles. As such, these two advanced imaging modalities are often seen as mutually exclusive. The primary aim of this study was to compare contrast-enhanced STE parameters with those from conventional unenhanced TTE. The secondary aim was to determine the impact of contrast-enhanced imaging on an inexperienced reader compared to an experienced reader of STE.

## Methods

### Ovine ECMO model

This research was performed at the Medical Engineering Research Facility at The Prince Charles Hospital, Brisbane, Australia. Approval had been obtained from the Animal Ethics Committee of the Queensland University of Technology (Approval no.1100000053) and the University of Queensland (Approval no. 194/12). Unenhanced and contrast-enhanced echocardiographic imaging was performed in our validated veno-venous (VV) ECMO ovine model. This research conformed to the National Health and Medical Research Council (NHMRC) Code of Practice for the Care and Use of Animals for Scientific Purposes (22). Anaesthetised sheep (18-month-old ewes, weighing 40–45 kg) were commenced on VV ECMO via access (22 French) and return (19 French) cannulae inserted in their right internal jugular vein (IJV). This ovine ECMO model has been described in detail previously (23). The sheep were supported with an ECMO circuit (Maquet Cardiopulmonary AG), consisting of Bioline tubing, Carmeda BioActive Surface-coated venous cannulae (Medtronic, MN, USA), a PLS Quadrox D oxygenator and a Rotaflow (Maquet, NJ, USA) pump head. A smoke-induced acute lung injury model was used in 21 of 24 sheep, prior to initiation of ECMO, using a validated and reproducible technique, as previously outlined (24). Briefly, this involved delivery of smoke via cotton combustion from manually compressed bellows until an arterial blood gas sample demonstrates a carboxyhaemoglobin level of 45–50%. VV ECMO alone was performed in three control sheep. VV ECMO was initiated using an infra-diaphragmatic inferior vena cava access cannula and a right atrial-superior vena caval region return cannula. 

### Transthoracic echocardiography

With the sheep in a sternal recumbent position, transthoracic echocardiography was performed by a single operator (DGP) in all sheep, using a Siemens Sequoia C512 scanner and 4V1 transducer. TTE imaging was performed following 20 h of ECMO support in 23 sheep and after 2 h of ECMO support in 1 sheep. Conventional parasternal short axis views, using ECG gating, were obtained initially. Due to the external morphology of a sheep chest wall, conventional apical TTE views cannot be obtained. 

### Contrast-enhanced transthoracic echocardiography

Immediately after the unenhanced TTE images were acquired, the contrast-enhanced TTE images were recorded. Activated Definity contrast (Lantheus Medical Imaging, Billerica, MA, USA) was diluted to 50 mL with normal saline and administered via an Alaris GH Plus infusion pump into an internal jugular central venous line. The infusion rate was varied to optimise image quality and was in the range 200–300 mL/h. A low mechanical index, proprietary imaging technique called Cadence Contrast Pulse Sequencing was used for all contrast-enhanced TTE images (25). The images were optimised to ensure a clear endocardial border, usually with adjustments in the mechanical index, gain settings and dynamic range. No flash destruction of the microbubbles was performed in this study.

### Echocardiographic image analysis

All images were transferred to a separate workstation and analysed using TomTec-Arena (TomTec Imaging Systems GMBH, Unterschleissheim, Germany). Unenhanced and then contrast-enhanced parasternal short axis (mid-left ventricular level) images were assessed. Following selection of an appropriate parasternal short axis clip, the endocardium at end diastole was tagged at three anatomic points, which then enabled the programme to automatically track endocardial motion. The epicardial border was automatically generated but could be manually adjusted. Veracity of tracking was then visually assessed and manually adjusted as required. In those images with poor tracking, analysis was not performed. Data collected were end diastolic area (EDA) (cm^2^), end systolic area (ESA) (cm^2^), fractional area change (FAC) (%), endocardial global circumferential strain (eGCS) (%), myocardial global circumferential strain (mGCS) (%), endocardial rotation (ER) (degrees, °) and global radial strain (GRS) (%). FAC was defined as the EDA minus the ESA divided by the EDA. eGCS was defined as the strain derived from the deformation of the endocardium in the circumferential direction in the left ventricular short axis. mGCS was defined as the strain derived from the deformation of the mid wall of the myocardium in the circumferential direction. ER was defined as the absolute rotation in the endocardium at the single defined parasternal short axis view (as opposed to myocardial rotation or torsion where basal and apical levels of myocardial analysis are required). GRS was defined as the strain derived from the whole thickness of the myocardium in the radial direction. This analysis was then repeated for a matching contrast-enhanced parasternal short axis image. All images were interpreted by two readers, one experienced in strain analysis (KJ) and one inexperienced in performing strain analysis (DGP). Each reader was blinded to the other reader’s analysis but the same unenhanced and contrast-enhanced images were assessed by each reader.

### Statistical analysis

Continuous variables are expressed as mean ± one standard deviation. Comparison between the continuous variables was performed using a paired *t* test. Inter-observer variability was assessed using the intra-class correlation coefficient (ICC). A *P* value of <0.05 was considered as statistically significant. Statistical analysis was performed using MedCalc version 10.0 (Mariakerke, Belgium).

## Results

Twenty-four sheep underwent unenhanced and contrast-enhanced TTE during VV ECMO and were included in this study. Of the 48 sheep datasets evaluated, three were excluded (two unenhanced and one contrast enhanced) due to poor endocardial tracking that resulted in negative FAC values. This resulted in a total of 22 sheep (92%) with paired evaluable unenhanced and contrast-enhanced images for final analysis. Mean ECMO flow during TTE imaging was 2.76 ± 0.58 L/min (range 1.46–3.79). Mean pump speed was 2668 ± 496 RPM (range 1460–3290). Mean contrast infusion rate was 247.9 ± 42.9 mL/h (range 200–300 mL/h). There was no significant difference between heart rate (98.8 ± 17.4 vs 103 ± 21.3 BPM) for unenhanced versus contrast-enhanced images respectively.) Frame rate for contrast-enhanced TTE was slower than unenhanced imaging (40 ± 8.4 vs 45 ± 12 Hz, *P* < 0.05).

**Figure 1:**
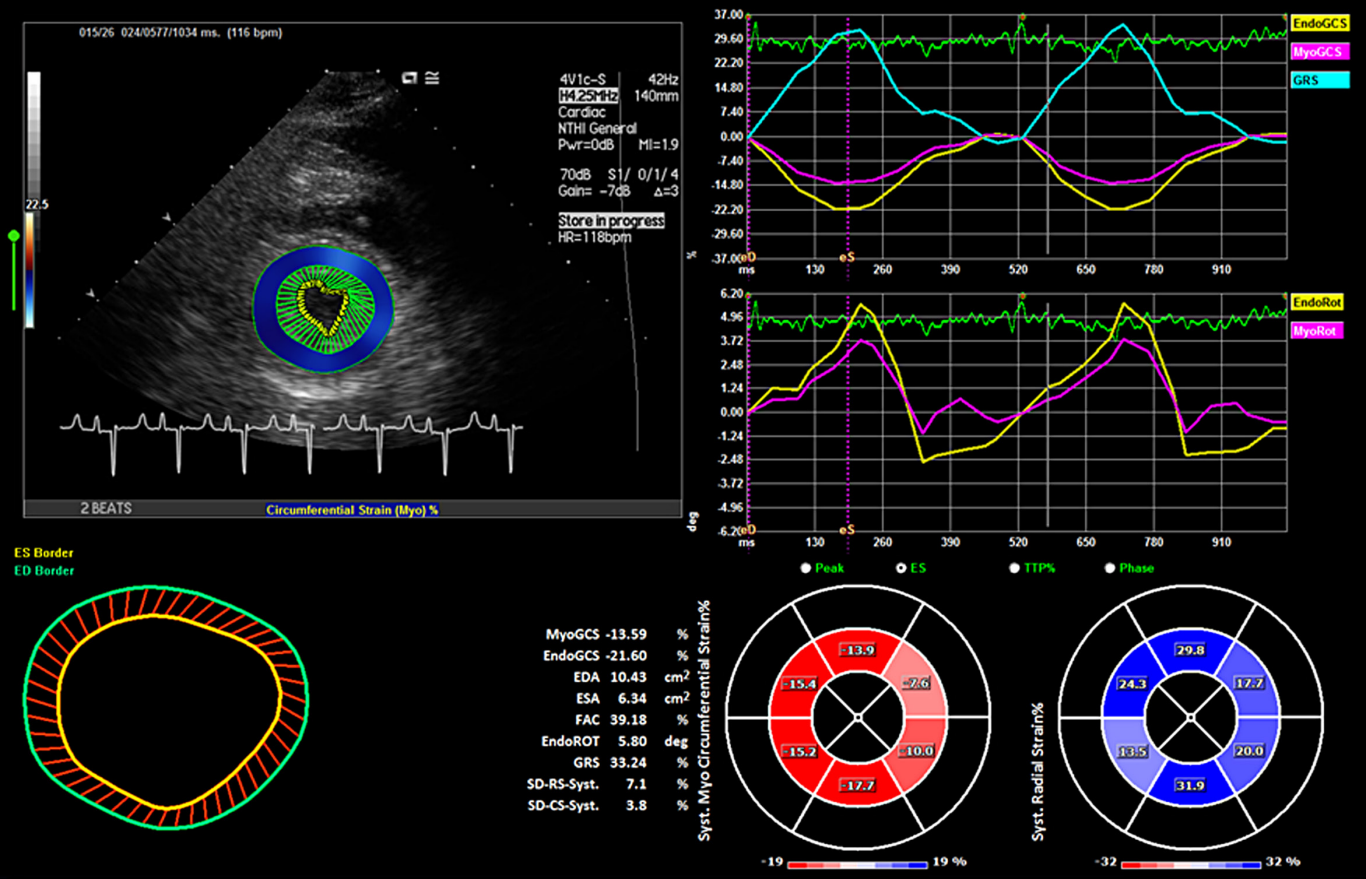
Analytical interface for unenhanced imaging in the parasternal short axis view, showing the EDA, ESA, FAC results (lower left panel) and the EGCS, MGCS, GRS and ER (upper right panels). Video 1 shows the corresponding clip.

**Figure 2:**
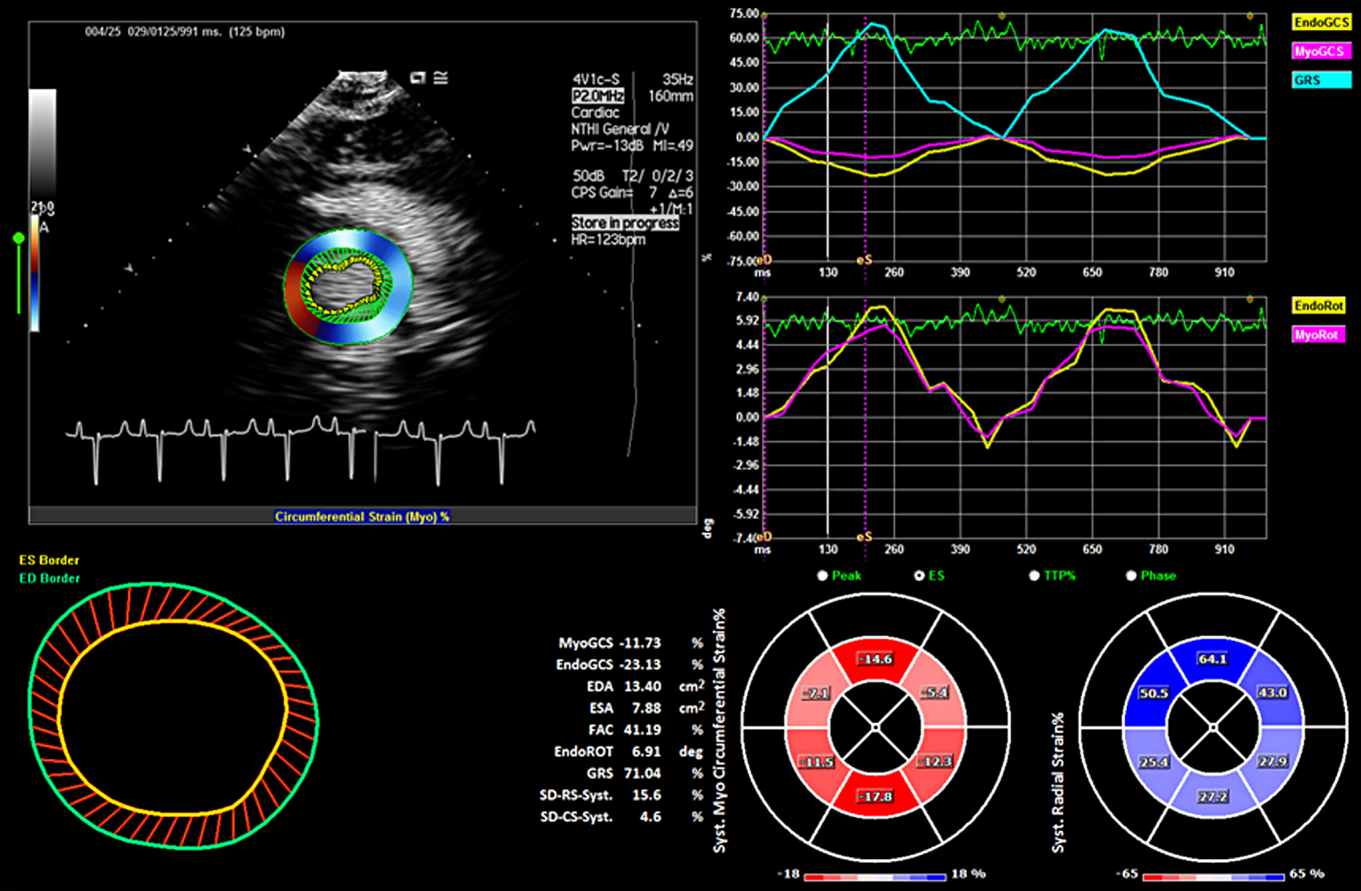
Analytical interface for contrast-enhanced imaging in the parasternal short axis view, showing the EDA, ESA, FAC results (lower left panel) and the EGCS, MGCS, GRS and ER (upper right panels). Reprinted from *Heart, Lung and Circulation*; vol **27**, Supplement 2; Platts D, Shiino K, Chan J, Burstow D, Scalia G & Fraser J; Comparison of unenhanced and contrast-enhanced echocardiographic assessment of myocardial function and mechanics during veno-venous extracorporeal membrane oxygenation; page S228; Copyright (2018), with permission from Elsevier. Video 2 shows the corresponding clip.

Results for the end diastolic area (cm^2^), end systolic area (cm^2^), fractional area change (%), endocardial global circumferential strain (%), myocardial global circumferential strain (%), endocardial rotation (degrees) and global radial strain (%), both for unenhanced and contrast-enhanced TTE by an experienced reader are shown in Table 1. The inter-observer variability (experienced versus inexperienced reader) for both unenhanced and contrast-enhanced TTE images are displayed in Table 2. Figures 1 and 2 are examples of the analytical interface for unenhanced and contrast-enhanced images respectively. Videos 1 and 2 are examples of endocardial tracking for unenhanced and contrast-enhanced images respectively.

**Table 1:** Mean ± 1 s.d. experienced reader unenhanced and contrast-enhanced TTE.

	Unenhanced TTE	Contrast enhanced TTE	*P*
EDA (cm^2^)	8.20 ± 2.31	11.75 ± 2.27	<0.0001
ESA (cm^2^)	4.45 ± 1.69	6.25 ± 1.64	<0.0001
FAC (%)	46.6 ± 8.82	46.98 ± 8.11	0.86
Endocardial GCS (%)	−26.69 ± 6.14	−26.88 ± 5.45	0.91
Myocardial GCS (%)	−17.52 ± 5.05	−16.51 ± 4.04	0.41
Endocardial rotation (degrees)	0.78 ± 6.14	1.23 ± 4.01	0.74
Global radial strain (%)	28.30 ± 12.08	69.00 ± 27.11	<0.0001

**Table 2:** Intra-class correlation coefficient for inter-observer variability, unenhanced versus contrast-enhanced TTE.

	Unenhanced TTE	95% CI	Contrast enhanced TTE	95% CI
EDA	0.54	0.13–0.78	0.89	0.75–0.95
ESA	0.58	0.16–0.82	0.85	0.68–0.94
FAC	0.70	0.39–0.87	0.88	0.72–0.95
Endocardial GCS	0.64	0.31–0.83	0.87	0.72–0.95
Myocardial GCS	0.48	0.06–0.75	0.75	0.48–0.89
Endocardial rotation	0.62	0.29–0.82	0.78	0.54–0.9
Global radial strain	0.55	0.19–0.78	0.71	0.43–0.93

Video 1 Example unenhanced imaging endocardial tracking. View Video 1 at http://movie-usa.glencoesoftware.com/video/10.1530/ERP-18-0071/video-1.
Download Video 1

Video 2 Example contrast-enhanced imaging endocardial tracking. View Video 2 at http://movie-usa.glencoesoftware.com/video/10.1530/ERP-18-0071/video-2.
Download Video 2

The mean contrast-enhanced EDA and ESA were significantly larger than the unenhanced images. However, the mean FAC was almost identical between the two techniques. There was no significant difference between unenhanced and contrast-enhanced mean endocardial global circumferential strain, myocardial global circumferential strain and endocardial rotation. However, there was a significant difference in global radial strain between the two techniques. Using the intra-class correlation coefficient, the inter-observer variability between the experienced and inexperience user was fair to good for unenhanced imaging (range 0.48–0.70) but good to excellent for the contrast enhanced images (range 0.71–0.89).

## Discussion

The main findings of this study are threefold. Firstly, semi-automated quantification of myocardial function and myocardial mechanics in the parasternal short axis view was technically feasible in the majority of unenhanced and contrast-enhanced images. Second mean fractional area change, endocardial global circumferential strain, myocardial circumferential strain and endocardial rotation were not significantly different between unenhanced and contrast-enhanced imaging. However, there were significant differences in end diastolic area, end systolic area and global radial strain. Third, the addition of contrast to the TTE imaging resulted in significantly less inter-observer variability, with the inexperienced reader results more closely approximating that of the experienced reader compared to unenhanced images.

Our research showed that unenhanced TTE areas (end diastolic and end systolic) were significantly less than contrast-enhanced areas but the final product (fractional area change) was similar. This finding is consistent with an extensive evidence base demonstrating that endocardial borders are better defined using contrast (26, 27, 28, 29). As a consequence of this, the true volumes or area is larger using contrast because ventricular trabeculation is excluded from the border measurement. Unenhanced imaging underestimates both volumes and areas as the trabeculation is usually included in the analysis as it tends to obscure the true endocardial border, where the correct measurement should be made. The addition of contrast significantly mitigates this underestimation of volumes or area in the clinical environment (30, 31, 32).

Circumferential stain in both the endocardium and myocardium was not significantly different between unenhanced and contrast-enhanced imaging in our study. In our study, circumferential strain was evaluated at both the endocardial and myocardial layers off the left ventricular wall. Circumferential strain, whilst not currently utilised in routine clinical practice, can be used to detect regional myocardial dysfunction and has been shown to be a useful predictor of prognosis in cardiac failure (33, 34, 35, 36). The left ventricular myo-architecture is complex and is macroscopically divided into the endocardium, mid-wall (thickest) and epicardial regions (37). The endocardial layer tends to have longitudinally orientated fibres whilst the thicker middle layer has more circumferentially orientated fibres (38, 39). 

Left ventricular rotation at the mid cavity level was assessed in our study. Rotation is defined as the rotational displacement around the long axis of the left ventricle and is measured in degrees (20). There was no significant difference between the mean unenhanced and contrast-enhanced mid cavity rotation. In isolation, this measurement is of limited clinical applicability. However, when combined with the rotational measurement at the left ventricular base and apex, the left ventricular twist angle can be calculated.

The results of our study showed that there was a significant difference between unenhanced versus contrast-enhanced global radial strain. There were very wide limits of agreement and the contrast-enhanced images provided significantly higher values than the unenhanced images. This wide variation in radial strain is a well-recognised limitation of this directional aspect of STE (40, 41). Our results are not surprising as other previous studies have also shown that the reproducibility of radial strain was the weakest in comparison to circumferential and longitudinal strain with the least consistency in measurements (42, 43). Strain can be measured in the longitudinal, circumferential and radial dimensions. Clinically, global longitudinal strain is well accepted and widely utilised. However, as variable involvement of the myocardium can occur with different disease states, other strain components may have a role to play. With the sub-endocardium being particularly sensitive to ischaemia, radial strain analysis may be of use in the detection of myocardial ischaemia (44, 45). However, in light of the method of acquisition and analysis, there are multiple technical factors that can cause this wide variation in radial strain (40). 

To our knowledge, there are few substantiated data in the literature that can confirm the feasibility of strain imaging during contrast administration. There is even less data on the evaluation of myocardial strain during ECMO. The calculation of myocardial strain parameters during contrast-enhanced echocardiography has traditionally been considered as not feasible with wide limits of agreement (46, 47). This may be due to tracking of a speckle from unenhanced myocardium being a more stable and static target than the stronger and more dynamic signal generated during contrast imaging. Despite both imaging modalities being advanced techniques, their method of image acquisition and interpretation, such as line density, signal-to-noise ratio and mechanical index, are fundamentally different. However, more recent work indicates that STE combined with contrast-enhanced imaging is feasible (48, 49, 50).

Accurate evaluation of cardiac function during a dynamic clinical course typical of ECMO support is fundamental in the management of such patients. For this to occur, clear identification of the cardiac structures, especially the endocardial borders is required. Ventricular function can be qualitatively measured using a semi-subjective visual evaluation. This typically grades ventricular function as normal, mild, moderately or severely impaired, along with a quoted estimate of ejection fraction. Systolic thickening of the myocardium is also an important component of evaluating myocardial function. Serial monitoring of ventricular function however requires more formal quantification of function (51, 52). 

Formal quantification of ventricular volumes and function can be performed using several methods. The conventional and most widely used technique is to calculate the ejection fraction using two dimensional TTE, usually by employing the stacked method of discs, or Simpson’s biplane method (18). This technique requires long axis imaging through the left ventricle in multiple planes. More recent advances in imaging technology have enabled accurate evaluation of the ejection fraction using three-dimensional echocardiography (53, 54, 55). All these qualitative and quantitative techniques rely on accurate identification of the endocardial border. Obtaining clear images of the endocardial border in the critical care setting however can be challenging and up to 25% of TTE images in this environment can be non-diagnostic. Factors that can prevent adequate TTE imaging include a supine ventilated patient, non-ideal lighting conditions and reduced acoustic windows. Contrast-enhanced TTE imaging can significantly improve visualisation of the endocardial border and convert a non-diagnostic set of images into a diagnostic scan (26, 56).

Contrast microspheres are hydrodynamically fragile structures and an ECMO circuit would represent an adverse environment for their stability and durability. The main sites for increased microsphere destruction are the oxygenator and within the ECMO pump rotor housing (57). Complex flow paths, high pressure changes and rapid turbulent flow all contribute to contrast destruction within an ECMO circuit. However, despite these ECMO factors adversely impacting on contrast microspheres, contrast-enhanced TTE has been shown to be feasible during ECMO support (58, 59, 60, 61). The increased contrast destruction can usually be overcome by increasing the infusion rate and hence maintaining adequate cardiac chamber opacification.

Our ECMO circuit utilised a ROTAFLOW (Maquet, NJ, USA) ECMO pump which did not have a built in venous bubble detector. Newer ECMO pumps, such as the CardioHelp (Maquet), have an integrated ultrasonic bubble detector. There has been some work to suggest that contrast microspheres may result in ECMO bubble detectors reading this as air within the circuit and critically impacting on ECMO circuit functionality (62). These ultrasonic bubble detectors are designed to detect air bubbles 0.3–0.5 mL in size (63). It is conceivable that due to the high backscattering properties of contrast microspheres, that this may mimic the presence of a large air bubble within the circuit, by similarly preventing clear and full transmission of a soundwave from the beam former to the receiver through the path of blood flow within the ECMO circuit tubing. In the clinical environment it is advised that staff should be aware of this possibility and anticipate a possible alarm with appropriate workflows in place for its management.

Evaluation of ventricular function has now progressed beyond just ejection fraction calculation. Myocardial deformation (which may be independent to ejection fraction) can be measured using simple tissue Doppler imaging or more advanced speckle tracking echocardiography (17, 18, 19). Global longitudinal strain has become a well-accepted, simple to perform, accurate and reproducible technique in the detection and monitoring of clinical and sub-clinical disease states (64, 65, 66, 67). Other parameters measured with speckle tracking echocardiography are also currently being assessed to determine clinical utility. These include circumferential and radial strain, rotation and torsion. However, like accurate quantification of ventricular volumes and ejection fraction, speckle tracking echocardiography requires good image quality, to enable tracking of the speckles. Poor image quality has traditionally thought to prevent accurate evaluation of these myocardial mechanic parameters. However, the results of this study add to the evidence base suggesting that contrast-enhanced imaging and speckle tracking echocardiography do not have to be mutually exclusive techniques and contrast enhanced imaging potentially improves the reproducibility of speckle tracking.

### Study limitations

This research was performed during ECMO support in a validated ovine model. These sheep do not have an apical window and as such, the clinically relevant and well-validated technique of global longitudinal strain could not be analysed. Whilst within the accepted workflow of our software programme, the frame rates were relatively low for STE and this may have had an impact on data analysis. Additionally, the frame rates for contrast-enhanced images were lower than with unenhanced imaging. Inter-vendor variability in measurement has been seen as a limitation of STE and work is on-going to determine the optimal work flow strategy to address this (68). Feasibility of quantification programmes in echocardiography require a metric about ease of use and measurement of analysis time. Formal timing of image analysis was not measured in our experiment. However, it was a relatively straight forward process that could usually be completed within 2–3 minutes per view. Finally, contrast imaging was performed regardless of baseline image quality. The majority had good endocardial definition. Hence the applicability of STE to contrast-enhanced imaging in those with non-diagnostic unenhanced images cannot be determined from this study.

## Conclusion

The results of our study show that semi-automated processing methods of myocardial function and mechanics using contrast-enhanced echocardiography during ECMO support is both feasible and similar to conventional unenhanced imaging for FAC, endocardial rotation and circumferential strain. Ventricular area measurement followed the well-recognised trend seen in the ventricular volume assessment, where unenhanced values were significantly lower than contrast enhanced values. Finally, the addition of contrast significantly decreased the inter-observer variability of all measurements. Translation of these results to the clinical environment suggest that in those patients supported with ECMO, contrast enhanced imaging in those with non-diagnostic echocardiograms may be a feasible technique for evaluation of myocardial function and deformation mechanics.

## Declaration of interest

David Platts is a Medical Liaison Officer for Lantheus Medical Imaging Australia. The other authors have nothing to disclose.

## Funding

This research was supported in part by funding from the National Health and Medical Research Council (grant no. 1010939) and The Prince Charles Hospital Foundation. John Fraser holds a Health Research Fellowship awarded by the Office of Health and Medical Research, Queensland, Australia. The Siemens Sequoia scanner used for this research was obtained with a research grant awarded to David Platts from the Private Practice Fund, The Prince Charles Hospital.

## Author contribution statement

D P designed the experiment, acquired the images, performed the analysis and composed the manuscript. K S performed analysis of the images and critically reviewed the manuscript. J C oversaw analysis using TomTec and critically reviewed the manuscript. D B and G M S critically reviewed the manuscript. J F F designed the ovine ECMO model and critically reviewed the manuscript. All institutional and national guidelines for the care and use of laboratory animals were followed.
